# T-cell gene therapy for perforin deficiency corrects cytotoxicity defects and prevents hemophagocytic lymphohistiocytosis manifestations

**DOI:** 10.1016/j.jaci.2017.11.050

**Published:** 2018-09

**Authors:** Sujal Ghosh, Marlene Carmo, Miguel Calero-Garcia, Ida Ricciardelli, Juan Carlos Bustamante Ogando, Michael P. Blundell, Axel Schambach, Philip G. Ashton-Rickardt, Claire Booth, Stephan Ehl, Kai Lehmberg, Adrian J. Thrasher, H. Bobby Gaspar

**Affiliations:** a Infection, Immunity, Inflammation, Molecular and Cellular Immunology Section, UCL Great Ormond Street Institute of Child Health, London, United Kingdom; b Department of Pediatric Oncology, Hematology and Clinical Immunology, Medical Faculty, Center of Child and Adolescent Health, Heinrich-Heine-University, Dusseldorf, Germany; c Institute of Experimental Hematology, Hannover Medical School, Hannover, Germany; d Section of Immunobiology, Division of Inflammation and Immunology, Department of Medicine, Faculty of Medicine, Imperial College, London, United Kingdom; e Center for Chronic Immunodeficiency, University Medical Center, University of Freiburg, Freiburg, Germany; f Department of Paediatric Haematology and Oncology, Division of Paediatric Stem Cell Transplantation and Immunology, University Medical Centre Hamburg-Eppendorf, Hamburg-Eppendorf, Germany

**Keywords:** Gene therapy, hemophagocytic lymphohistiocytosis, perforin deficiency, T cells, FHL, Familial hemophagocytic lymphohistiocytosis, GFP, Green fluorescent protein, HLH, Hemophagocytic lymphohistiocytosis, HSCT, Hematopoietic stem cell transplantation, IRES, Internal ribosomal entry site, LCMV, Lymphocytic choriomeningitis virus, NK, Natural killer, PRF1, Perforin 1, PS, Penicillin and streptomycin, TCM, Central memory T, TEM, Effector memory T, WT, Wild-type

## Abstract

**Background:**

Mutations in the perforin 1 *(PRF1)* gene account for up to 58% of familial hemophagocytic lymphohistiocytosis syndromes. The resulting defects in effector cell cytotoxicity lead to hypercytokinemia and hyperactivation with inflammation in various organs.

**Objective:**

We sought to determine whether autologous gene-corrected T cells can restore cytotoxic function, reduce disease activity, and prevent hemophagocytic lymphohistiocytosis (HLH) symptoms in *in vivo* models.

**Methods:**

We developed a gammaretroviral vector to transduce murine CD8 T cells in the *Prf*^−/−^ mouse model. To verify functional correction of *Prf*^−/−^ CD8 T cells *in vivo*, we used a lymphocytic choriomeningitis virus (LCMV) epitope–transfected murine lung carcinoma cell tumor model. Furthermore, we challenged gene-corrected and uncorrected mice with LCMV. One patient sample was transduced with a *PRF1*-encoding lentiviral vector to study restoration of cytotoxicity in human cells.

**Results:**

We demonstrated efficient engraftment and functional reconstitution of cytotoxicity after intravenous administration of gene-corrected *Prf*^−/−^ CD8 T cells into *Prf*^−/−^ mice. In the tumor model infusion of *Prf*^−/−^ gene–corrected CD8 T cells eliminated the tumor as efficiently as transplantation of wild-type CD8 T cells. Similarly, mice reconstituted with gene-corrected *Prf*^−/−^ CD8 T cells displayed complete protection from the HLH phenotype after infection with LCMV. Patients' cells showed correction of cytotoxicity in human CD8 T cells after transduction.

**Conclusion:**

These data demonstrate the potential application of T-cell gene therapy in reconstituting cytotoxic function and protection against HLH in the setting of perforin deficiency.

After viral or other infections, activated effector T and natural killer (NK) cells form an immunologic synapse with target cells and release specialized granules that mediate cell cytotoxicity. Mutations in genes that encode key components of the CD8 T cell and NK cell cytolytic pathway^1^, ^2^, ^3^ lead to defects of cytotoxic function and failure of elimination of infected and malignant cells. Persistent antigen presentation results in hypercytokinemia and immune dysregulation with macrophage activation and leads to a condition known as hemophagocytic lymphohistiocytosis (HLH). Cardinal features of HLH include prolonged fever, hepatosplenomegaly, cytopenia, and various laboratory abnormalities, including decreased NK cell activity and increased ferritin levels.

The first of these genes to be identified was perforin 1 (*PRF1*; familial hemophagocytic lymphohistiocytosis [FHL] 2).^4^ The protein is contained within exocytic granules and, upon release into the immunologic synapse, forms pores on the surface of target cells. Subsequently it allows the passage of granzymes into the cytoplasm of target cells, thereby initiating apoptotic pathways and eventually leading to target cell death.^5^ Mutations in human *PRF1* account for up to 58% of FHL cases depending on ethnicity.^1^

Management of HLH encompasses combination chemotherapy, serotherapy, or both to suppress immune activation before definitive therapy with allogeneic hematopoietic stem cell transplantation (HSCT).^6^, ^7^, ^8^, ^9^, ^10^, ^11^ In 20% of cases, HLH does not respond to conventional treatment, and patients die of overwhelming immune dysregulation. Once in clinical remission, optimal donor availability and reduced-intensity conditioning can deliver a survival rate of up to 90%, but in patients with incomplete clinical remission and in the context of a mismatched donor, survival is less than 50%.^12^, ^13^

A murine perforin-deficient model of HLH has been generated that accurately recapitulates the immunologic characteristics of the disease^14^ after lymphocytic choriomeningitis virus (LCMV) challenge, and furthermore, *Prf*^−/−^ mice are unable to reject transplanted tumors. Correction of immune dysregulation in this model by means of transplantation with wild-type (WT) bone marrow shows that prevention of HLH development after LCMV infection is critically dependent on engraftment of functional CD8 T cells.^15^

Previously, we have shown in a murine *Prf*^−/−^ model that transplantation of *PRF1* gene–corrected progenitor cells results in expression of perforin in T and NK cells and leads to significant correction of cytotoxic defects both *in vitro* and *in vivo*.^16^ One concern with this approach is that perforin expression in progenitor cells is not physiologic and can result in stem cell dysfunction. Although no adverse events were seen, we wanted to evaluate whether transfer of gene-modified T cells and specifically CD8 T cells could correct the disease phenotype, especially because functional CD8 T cells are critical determinants of disease protection.^14^, ^15^, ^17^ The use of autologous gene-modified T cells also has an established safety profile, with hundreds of patients treated to date for hematologic malignancies in cancer immunotherapy trials with no reported transformational events.

In this study we show that transplantation of gene-corrected CD8 T cells leads to functional reconstitution of the cytotoxic defect and allows successful tumor clearance. Similarly, *Prf*^−/−^ mice reconstituted with gene-corrected CD8 T cells were protected from the features of HLH after infection with LCMV. Finally, we were able to transduce human PBMCs from perforin-deficient patients and correct the cytotoxic defect *in vitro*. Our results suggest that gene addition into autologous CD8 T cells might be a useful therapeutic approach for perforin-deficient HLH.

## Methods

### Mouse/human samples

Perforin-deficient (*Prf*^−/−^;C57BL/6-Prf1tm1Sdz/J) and P14 (B6;D2-Tg[TcrLCMV]327Sdz/JDvsJ, transgenic T-cell receptor specific for LCMV-derived GP33 epitope) mice were obtained from the Jackson Laboratory (Bar Harbor, Me). C57BL/6 and C57BL/6-Ly5.1 (B6;WT) mice were bred at our facility, and P14 mice deficient in perforin (P14 *Prf*^−/−^) were generated by breeding with *Prf*^−/−^ mice. Donor and recipient mice were usually between 8 and 16 weeks of age in all experiments. All experiment procedures were approved by the Institutional Research Ethics Committee (Great Ormond Street Institute of Child Health, University College London and Imperial College, London, United Kingdom). All murine experiments were performed according to UK Home Office Animal Welfare Legislation. Please refer to the Methods section in this article's Online Repository at www.jacionline.org for detailed treatment and numbers of mice used in each investigated group. Healthy donor and patient samples were acquired from the Great Ormond Street Hospital and collaborators.

### Vector construction

RV PRF, a retroviral vector incorporating perforin cDNA, an internal ribosomal entry site (IRES) element, and green fluorescent protein (GFP), was constructed. The native human perforin gene and IRES and GFP sequences were removed by means of enzyme restriction from a lentiviral PGK.PRF.IRES.GFP vector previously described^16^ and cloned into an SF91.GFP backbone after removal of the original GFP sequence. The backbone was a kind gift from Christopher Baum (Hannover, Germany)^18^; in this original vector the GFP is controlled by the RV SFFV long terminal repeat.

### Tumor cell line/LCMV

#### A9GP33 cells

We used an immunogenic and low metastatic cloned line derived from 3LL Lewis lung carcinoma, which was transfected by the LCMV GP33 miniepitope (cell lines were kindly provided by Hanspeter Pircher, Freiburg, Germany).^19^ A9GP33 tumor cells were cultivated with Dulbecco modified Eagle medium supplemented with 10% FCS, 1% penicillin and streptomycin (PS), and Genetecin (800 μg/mL; Thermo Fisher Scientific, Waltham, Mass) for selection purposes (all from Gibco Life Technologies, Carlsbad, Calif). Mice were injected subcutaneously in the neck after achievement of inhalational anesthesia with isoflurane. A9GP33 tumor cells (10^7^) were applied in a volume of 200 μL (1:1 Dulbecco modified Eagle medium and Matrigel ECM Matrix [Corning, Corning, NY]). Tumor size and presence of a palpable tumor were evaluated daily, and tumor size was determined with a caliper. Mice bearing a tumor with a diameter of greater than 10 mm were culled according to animal care and welfare regulations.

#### LCMV

Mice were infected with 10^5^ plaque-forming units of LCMV Armstrong by means of intraperitoneal injection 4 weeks after CD8 T cell transfer. All mice were bled on day 8 after infection. They were killed if severe clinical signs of HLH/continuous weight loss developed before mice died during the natural course of disease to meet animal license regulations. Mice were checked for weight loss (15% was the threshold for immediate culling), hunched posture, ruffled hair, reduced mobility, reduced strength, reduced interaction with cage mates, and increased respiratory effort (according to the animal license) by staff of the animal facility. These staff were blind to individual treatment groups of mice and therefore were able to assess the clinical state of the mice in an unbiased manner. Based on clinical parameters, mice were either culled or allowed to continue to the end point of the experimental protocol. Mice undergoing transplantation were monitored for a maximum period of another 4 weeks.

### CD8 T cell stimulation and transduction

Murine splenocytes were harvested (day 1), and CD8 T cells were isolated by means of positive magnetic selection (CD8a [Ly-2] MicroBeads; Miltenyi Biotec, Bergisch Gladbach, Germany). CD8 T cells were cultured in RPMI 1640, 10% FCS, 1% PS, 1 mmol/L β-mercaptoethanol, and 1 mmol/L sodium pyruvate (all from Life Technologies) and stimulated with 50 U/mL murine IL-2 (PeproTech, Rocky Hill, NJ) and 2.5 μg/mL purified hamster anti-mouse CD3e (BD Biosciences, San Jose, Calif). Transduction was performed 24 hours later with retroviral supernatant through spinoculation (90 minutes at 1000*g*) in recombinant human fibronectin fragment (RetroNectin; Takara Bio Europe S.A.S, Saint-Germain-en-Laye, France)–precoated plates. Transduction efficiency was measured *in vitro* on day 5. CD8 T cells (5 × 10^6^-10^7^) were transplanted on day 3 by means of intravenous tail vein injection into *Prf*^−/−^ mice either sublethally (6 Gy) irradiated or injected with tumor cells (see above). Human PBMCs were isolated by means of Ficoll-Paque isolation and stimulated with CD3/CD28 Dynabeads (Gibco, Life Technologies) and human 100 U/mL IL-2 (100 U/mL; PeproTech). Twenty-four to 48 hours later, cells were transduced with the lentiviral vector at a multiplicity of infection of 25 to 100. Perforin and GFP expression was measured after another 72 hours, according to the manufacturer's protocol (BD Biosciences). Flow cytometric antibodies were purchased from Miltenyi Biotec or BD Biosciences. Sample analyses were performed with a CyAn ADP Analyzer (DAKO, Santa Clara, Calif) and FlowJo software (Version X; TreeStar, Ashland, Ore).

### Cytotoxicity

#### P815 cells

The murine mastocytoma cell line P815 (ATCC, Manassas, Va) serves as a classical target for T-cell cytotoxicity in both human subjects and mice. For the CD8 T cell–redirected killing assay, murine CD8 T lymphoblasts (isolated from spleens and stimulated for 48 hours, as described above) conjugated with anti-CD3 (BD Biosciences) and ^51^Cr-labeled (Na_2_^51^CrO_4_; PerkinElmer, Waltham, Mass) P815 target cells were mixed in 96-well round-bottom plates at various effector/target ratios and incubated for 4 hours at 37°C. Human CD8 T lymphoblasts were also targeted against P815 cells 7 days after transduction. For antigen-specific CD8 T cell function, P14 *Prf*^−/−^ splenocytes were cultured in RPMI1640 (10% FCS, 1% PS, and 50 mmol/L β-mercaptoethanol) and stimulated with LCMV gp33 peptide (KAVYNFATM; iba, Goettingen, Germany; 10^−7^ mol/L gp33, 100 ng/mL) for 48 hours and murine IL-2 (100 U/mL; PeproTech) for a further 72 hours. ^51^Cr release in the supernatant was measured with a beta counter (1450 MicroBeta TriLux; PerkinElmer). All assays were done in triplicates.

#### IFN-γ release assay

CD8 T lymphoblasts (1 × 10^5^; stimulated for 48 hours) labeled with 1 μg/mL anti-CD3 and 5 × 10^3^ P815 target cells were mixed in 96-well round-bottom plates and incubated for 4 hours at 37°C. IFN-γ concentrations were determined in supernatants by means of ELISA (Mouse IFNg “Femto-HS” High Sensitivity ELISA Ready-SET-Go; eBioscience, San Diego, Calif).

### Statistics

Plots were generated with Excel 2011 (Microsoft, Redmond, Wash) or GraphPad Prism (6.0; GraphPad Software, La Jolla, Calif) software. The Mann-Whitney *U* (Wilcoxon rank sum) test (IFN-γ levels and GFP expression), Student *t* test, and 2-way ANOVA (tumor growth and cytotoxicity) were applied to calculate significance.

## Results

### Gammaretroviral murine CD8 T cell perforin gene transfer restores cytotoxicity *in vitro*

**Fig 1:**
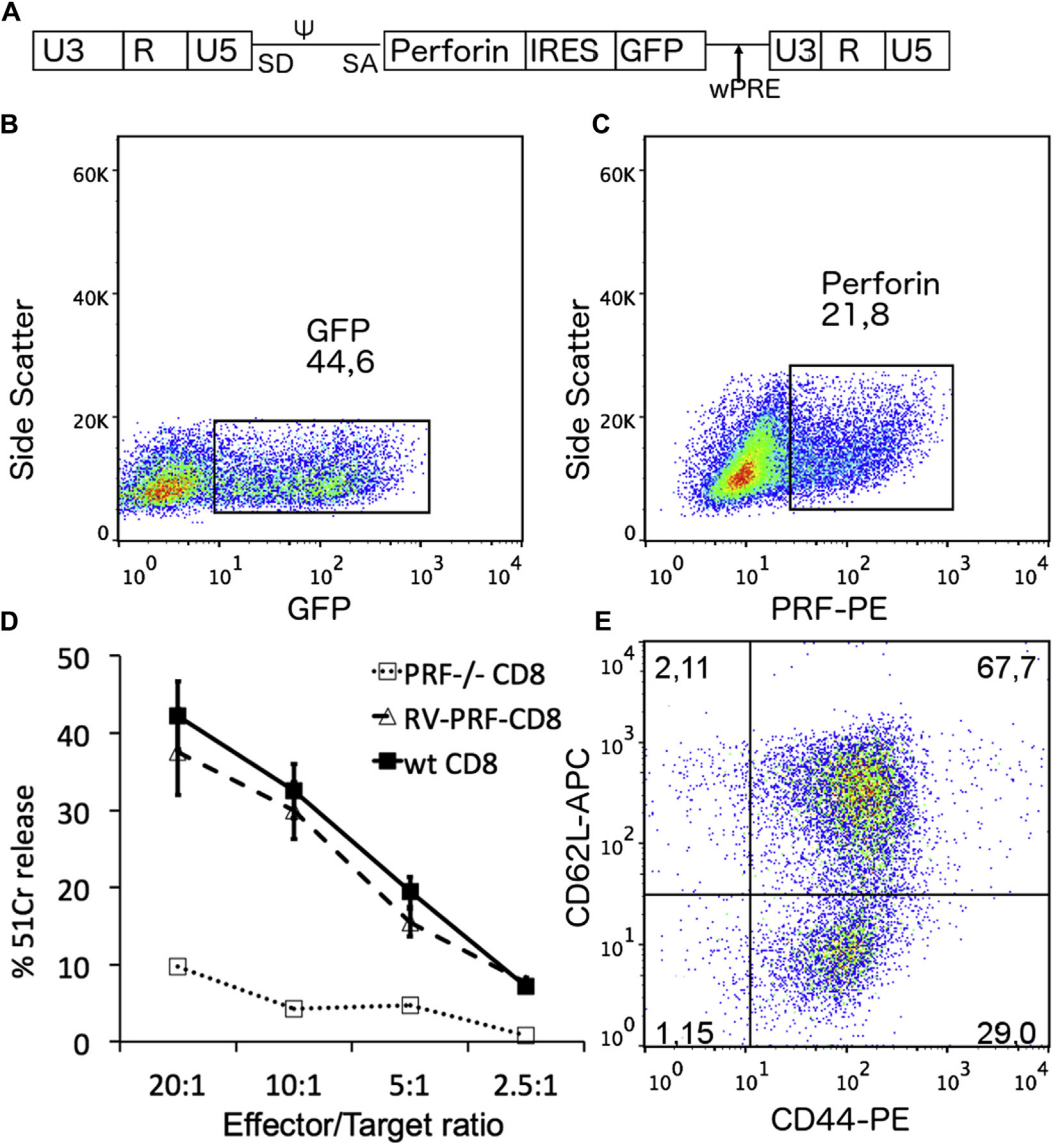
*In vitro* transduction of *Prf*^−/−^ CD8 T cells. **A,** An SF91 gammaretroviral vector *(RV PRF)* containing the spleen focus–forming viral long terminal repeat *(LTR)* and the woodchuck hepatitis virus posttranscriptional regulatory element *(WPRE)* encoding GFP and human perforin was constructed to transduce murine CD8 T cells. **B** and **C,** Transduction of isolated murine CD8 T cells with retroviral supernatant leads to GFP expression of between 40% and 50% and expression of human perforin of between 15% and 30%. **D,** A redirected cytotoxicity assay against P815 target cells shows complete restoration of RV-PRF–transduced *Prf*^−/−^ CD8 T cells *(RV-PRF-CD8)* similar to WT CD8 T cells *(wt CD8)*. Untransduced *Prf*^−/−^ CD8 T cells show low cytotoxicity *(PRF*^*-/-*^*CD8)*. Data are shown as means ± SDs. Two-way ANOVA calculated statistical significant difference between WT/corrected versus uncorrected CD8 T cells. **E,** Stimulation of isolated murine CD8 T cells leads to a predominantly central (CD62L^+^CD44^+^, *upper right quadrant*) or effector (CD62L^−^CD44^+^, *lower right quadrant*) memory CD8 T-cell phenotype. *APC*, Allophycocyanin; *PE*, phycoerythrin.

**Fig E1:**
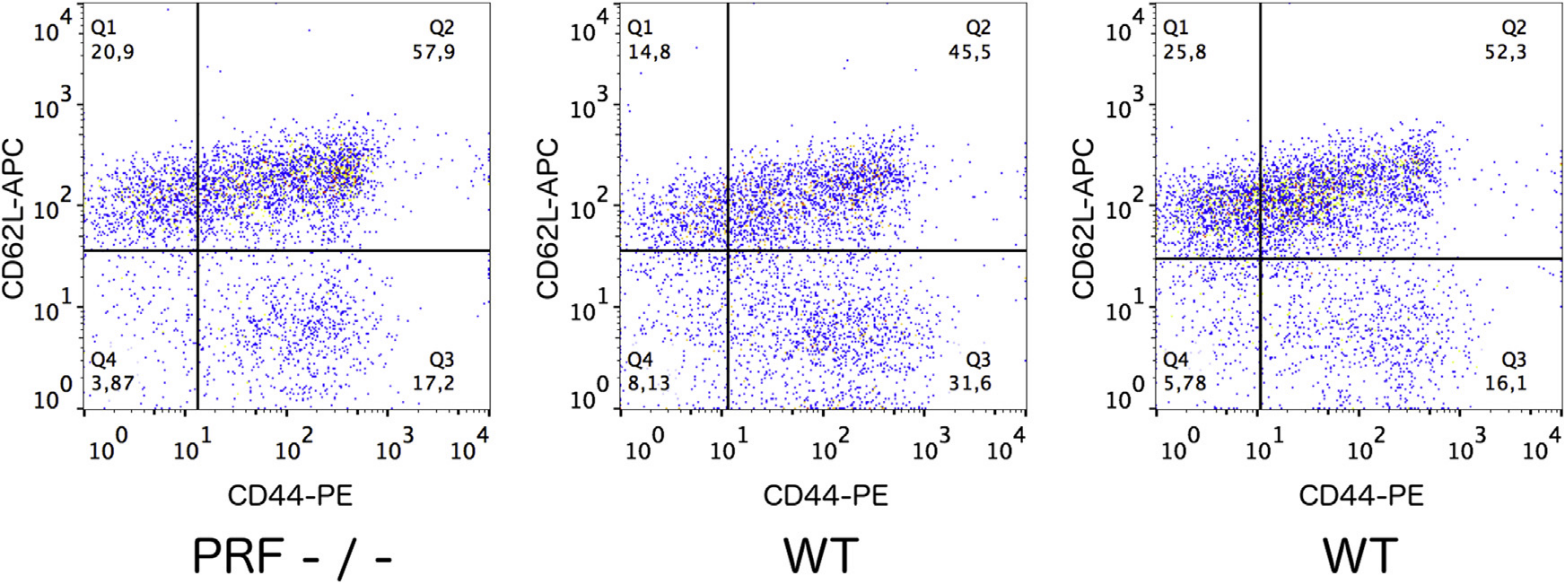
Baseline investigations of splenic CD8 T cells in both *Prf*^−/−^*(left panel)* and WT B6 (*middle* and *right panels*) mice show higher numbers of CD8 stem T cells (CD62L^+^CD44^−^) than in stimulated cells (Fig 1, *E*), which led to a predominantly central (CD62L^+^CD44^+^, *upper right quadrant*) or effector (CD62L^−^CD44^+^, *lower right quadrant*) memory CD8 T cell phenotype. *APC*, Allophycocyanin; *PE*, phycoerythrin.

A bicistronic gammaretroviral vector (Fig 1, *A*) encoding *PRF1* and a linked Gfp cDNA was generated and able to transduce CD8 T cells effectively, with Gfp and perforin expression of 45% and 21%, respectively (Fig 1, *B* and *C*). This difference is attributable to the increased detection sensitivity of the Gfp protein and has been seen with other constructs. Three to 5 days after transduction, cytotoxicity was measured in a Cr^51^ release assay (redirected killing of P815 cells with anti-CD3–conjugated CD8 T cells). Correction of *Prf*^−/−^ CD8 T cells with the gammaretroviral vector led to cytotoxicity results similar to those seen in WT CD8 T cells (Fig 1, *D*). As expected, the cytokine stimulation necessary to allow efficient gammaretroviral transduction led to a more differentiated CD8 T cell phenotype, and the majority of both GFP-negative and GFP-positive cells were of a central memory T (TCM) and effector memory T (TEM) phenotype (gating strategy as shown elsewhere^20^; Fig 1, *E*, and see Fig E1 in this article's Online Repository at www.jacionline.org for comparison with mice before stimulation/transduction).

### Transfer of gene-corrected *Prf*^−/−^ CD8 T cells corrects CD8 T cell cytotoxicity in *Prf*^−/−^ mice

**Fig 2:**
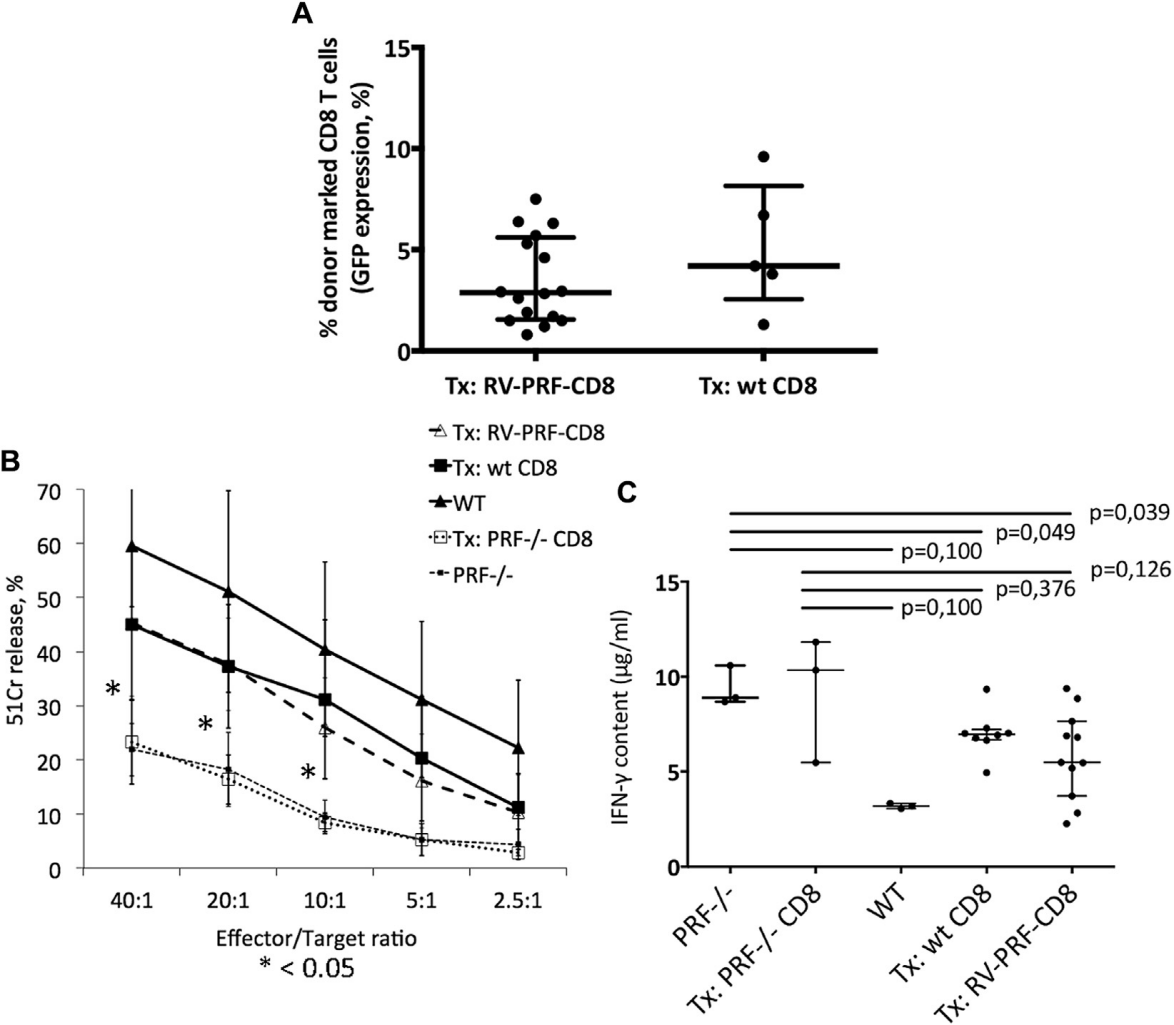
Reconstitution of the CD8 T cell compartment in *Prf*^−/−^ mice after adoptive transfer of RV-PRF–transduced *Prf*^−/−^ CD8 T cells. **A,** Engraftment was measured based on GFP expression. Both cohorts show similar engraftment levels without a significant difference. **B,** Cytotoxicity assay of reconstituted *Prf*^−/−^ or WT mice with P815 cells. Data are shown as means ± SDs of all investigated animals. Two-way ANOVA was used to calculate statistically significant differences between the WT and untreated groups, as well as the treated versus untreated groups. There is no significant difference between both interventional groups (wt CD8 vs RV-PRF-CD8). *Asterisks* indicate a *P* value of less than .05 between the treated versus untreated groups. **C,***In vitro* IFN-γ production measured in supernatants after coincubating splenic CD8 T cells with P815 cells. *Left* to *right*, *Prf*^−/−^ mice, *Prf*^−/−^ mice transplanted with uncorrected *Prf*^−/−^ CD8 T cells, WT mice, *Prf*^−/−^ mice receiving adoptive transfer of WT CD8 T cells, and *Prf*^−/−^ mice receiving RV-PRF–transduced *Prf*^−/−^ CD8 T cells. *Symbols* in Fig 2, *A* and *C*, represent individual mice in each treatment group. The *horizontal bar* represents the median, and *whiskers* mark the interquartile range.

We then determined whether adoptive T-cell transfer rescues the cytotoxic defect in *Prf*^−/−^ mice. Because the immunopathology in *Prf*^−/−^ mice clearly relates to a CD8 T cell dysfunction,^14^ we studied the transfer of either WT or *PRF1* gene-corrected *Prf*^−/−^ splenic CD8 T cells into *Prf*^−/−^ mice. Three weeks after transplantation into sublethally irradiated mice, in both the WT and perforin-transduced cohorts, the donor CD8 T cell population in peripheral blood consisted of a mean of 12% donor marked cells. At a preselected time of 2 months after transplantation, mice were culled, and peripheral blood and splenocytes were analyzed for donor marking. Engraftment based on GFP measurement was similar in both cohorts (Fig 2, *A*) at a mean of 3.5% in the gene-corrected cohort and 5.1% in the WT transplanted group. Cytotoxicity assays showed rescue of CD8 T cell cytotoxicity in both WT CD8 T-cell and RV PRF CD8 T cell transplants at levels statistically significantly increased compared with that seen in negative controls (*Prf*^−/−^ mice and *Prf*^−/−^ mice receiving GFP only–transduced CD8 T cells), although recovery did not reach values seen in untransplanted WT mice (Fig 2, *B*). Equally, IFN-γ release on targeting CD8 T cells with P815 cells led to decreased IFN-γ levels in both WT and RV PRF–treated mice in comparison with negative control animals (Fig 2, *C*), although the difference was statistically not significant because of a low sample number. However, we did not observe any correlation between IFN-γ levels and GFP expression in CD8 T cells in gene-corrected mice (data not shown).

### Transfer of gene-corrected *Prf*^−/−^ CD8 T cells leads to protection against tumor growth in an antigen-specific tumor model both *in vitro* and *in vivo*

**Fig 3:**
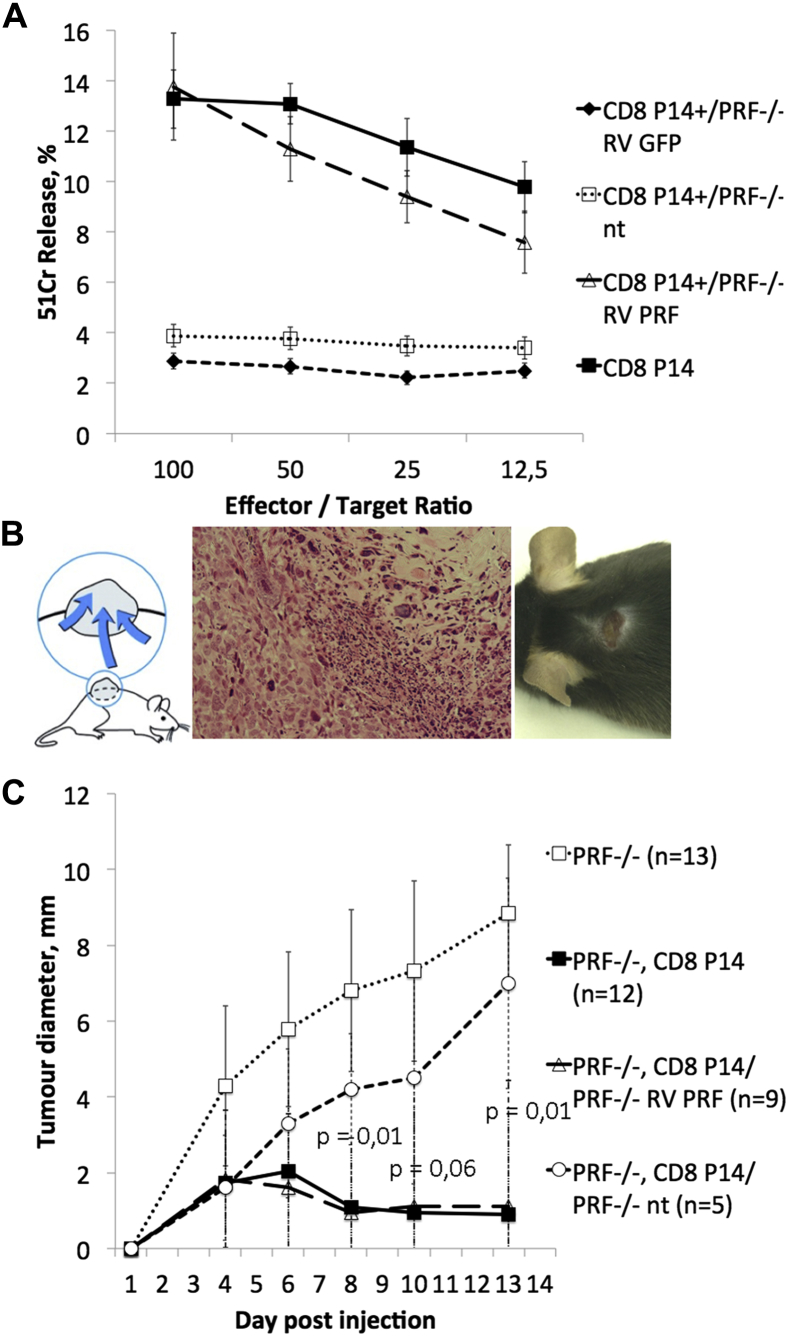
Tumor model for antigen-specific challenge of CD8 T cells. **A,** Antigen-specific cytotoxicity was measured in a cytotoxicity assay targeting A9GP33 cells. RV-PRF–transduced P14^+^/*Prf*^−/−^ CD8 T cells show equal cytotoxicity similar to P14 CD8 T cells, whereas uncorrected P14^+^/*Prf*^−/−^ CD8 T cells show absent cytotoxicity. Data are shown as means ± SDs. Two-way ANOVA was used to calculate significant differences between both WT and corrected versus uncorrected CD8 T cells. **B,***Left* and *right*, Subcutaneous injection of A9GP33 cells into the neck leads to formation of a localized tumor. *Middle panel*, Histology of tumor in hematoxylin and eosin stain and ×100 magnification. **C,** Tumor growth in *Prf*^−/−^ mice. Untreated *Prf*^−/−^ mice or *Prf*^−/−^ mice receiving adoptive CD8 T cell transfer from perforin-deficient P14 (P14^+^/*Prf*^−/−^) mice did not suppress tumor growth. In mice receiving P14 or RV-PRF–transduced P14^+^/*Prf*^−/−^ CD8 T cells at the time of tumor cell injection, formation of the tumor is stopped at an early stage. Data are shown as means ± SDs of all investigated animals.

We used a murine lung carcinoma tumor model (A9) expressing the LCMV GP33 epitope (A9-GP33 tumor line) to challenge the antigen-specific cytotoxic function of gene-corrected T cells *in vivo* and *in vitro*. P14 mice (transgenic GP33-specific T-cell receptor) are not susceptible to tumor formation. Therefore we crossed P14 mice with *Prf*^−/−^ mice to generate an *in vivo* model of defective cytotoxicity and verified this by using A9GP33 cells as targets. CD8 T cells from P14 *Prf*^−/−^ mice transduced with the gammaretroviral perforin vector showed full recovery of cytotoxicity similar to that seen in CD8 T cells from P14 mice (Fig 3, *A*). P14 *Prf*^−/−^ splenocytes transduced with an empty GFP-expressing vector exhibited absent cytotoxicity similar to that of untransduced cells. To assess these findings *in vivo*, we injected the A9G33 line subcutaneously into *Prf*^−/−^ mice to induce tumor development (Fig 3, *B*). These mice received adoptive transfer of either WT P14 or P14 *Prf*^−/−^ CD8 T cells (with and without retroviral transduction with RV PRF) on the same day. In serial experiments transduction efficiency was 28% to 47%, which did not change the clinical outcome. Tumor development was assayed with a caliper. All mice receiving WT P14 cells or successfully transduced P14 *Prf*^−/−^ CD8 T cells eliminated the tumor, whereas in untreated *Prf*^−/−^ mice or *Prf*^−/−^ mice transplanted with untransduced P14 *Prf*^−/−^ CD8 T cells, tumor growth exceeded set limits (10 mm) or erupted, and therefore mice were humanely culled (Fig 3, *C*). Two-way ANOVA calculated the statistically significant difference between mice harboring WT or corrected CD8 T cells versus mice with an uncorrected *Prf*^−/−^ gene.

### Transfer of gene-corrected *Prf*^−/−^ CD8 T cells prevents LCMV induced HLH-like disease

Because of the cytotoxic defect, *Prf*^−/−^ mice typically experience an HLH-like clinical phenotype with progressive cytopenia, hepatosplenomegaly, and hyperinflammation after LCMV infection. We investigated whether transfer of *PRF1* gene–corrected CD8 T cells could protect against LCMV infection. *Prf*^−/−^ mice were transplanted with WT CD8 T cells, RV PRF–corrected CD8 T cells, or GFP-transduced CD8 T cells after sublethal irradiation. Transduction efficiency was between 27% and 36% in serial experiments without any correlation to clinical outcome. Four weeks later, before LCMV infection, we saw an engraftment of 8% to 15% (measured based on GFP expression) of gene-modified CD8 T cells in peripheral blood in the RV PRF CD8 T cell group, as seen in previous experiments (data not shown).

**Fig 4:**
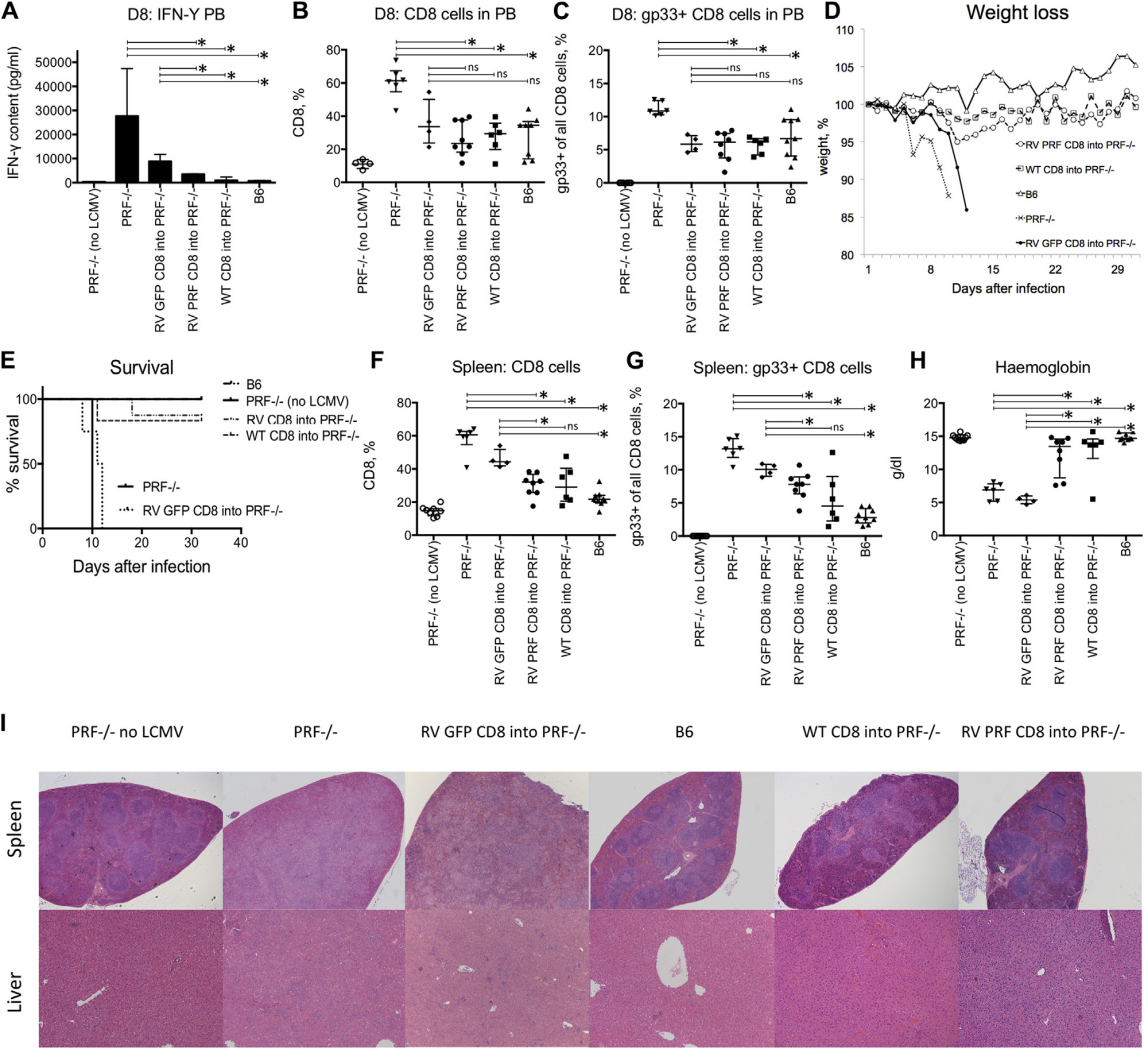
LCMV infection after CD8 T cell transduction. Transfer of corrected or WT CD8 T cells prevents mice from development of the HLH-like phenotype. CD8 T cells were transplanted into *Prf*^−/−^ mice. Four weeks later, transplanted mice and control animals were infected with LCMV. **A-C,** Serum IFN-γ level (Fig 4, *A*) and phenotype (Fig 4, *B* and *C*) of PBMCs were assessed in infected and uninfected *Prf*^−/−^ mice on day 8. **D,** Weight loss in untreated *Prf*^−/−^ mice after LCMV infection. **E,** Survival was monitored until day 30 and is depicted as a Kaplan-Meier curve. All infected *Prf*^−/−^ mice without prior transplantation and RV GFP CD8 cell–transplanted mice were culled between days 8 and 12. One mouse of each treatment group had HLH and had to be culled subsequently. **F** and **G,** Phenotype of splenocytes show an expansion of total CD8 T cells and gp33^+^ CD8 T cells in *Prf*^−/−^ mice, which is in line with the phenotype of PBMCs. Prior transplantation of corrected CD8 T cells shows a decrease in CD8/CD8 gp33 T-cell expansion. **H,** Hemoglobin levels in treated compared with untreated mice. Data are shown as medians ± interquartile ranges of all investigated animals. *P < .05. *ns*, Nonsignificant. **I,***Upper panel*, Hematoxylin and eosin–stained splenic sections show deranged splenic architecture in *Prf*^−/−^ mice without corrective intervention, whereas in treated and B6 mice it is preserved. *Lower panel*, Hematoxylin and eosin–stained liver sections display inflammatory foci and periportal lymphocytic infiltrates in untreated infected mice, whereas treated mice show similar liver histology as uninfected mice. Representative sections are shown at a magnification of ×5 (spleen) and ×10 (liver).

Control mice, which were neither irradiated nor transplanted (WT B6, *Prf*^−/−^), and transplanted mice (4 weeks after transplantation) were then infected with 10^5^ plaque-forming units LCMV Armstrong administered intraperitoneally. On day 8 after LCMV infection, we observed increased IFN-γ serum levels and an increase in numbers of CD8 T cells, which was accompanied by a tetramer-specific expansion of cells against the LCMV gp33 epitope in *Prf*^−/−^ mice. However, low levels of IFN-γ production were seen in B6 mice, and similarly, low levels were seen in *Prf*^−/−^ mice transplanted with WT or *PRF1* gene–corrected CD8 T cells. By contrast, in *Prf*^−/−^ mice there was no CD8- or gp33-specific CD8 T-cell expansion. *Prf*^−/−^ mice transplanted with RV GFP–transduced CD8 T cells showed a reduction in IFN-γ levels, but this did not approach WT values and a level of CD8 and tetramer-specific T cells that were similar to WT values (Fig 4, *A-C*). We did not observe any correlation between IFN-γ levels and GFP expression in CD8 T cells in gene-corrected mice (data not shown).

All interventional mice received sublethal irradiation, which might confound CD8 T cell and IFN-γ results. All untreated or GFP only–treated *Prf*^−/−^ mice had to be culled between day 8 and day 12 because of the clinical course and weight loss, but the B6 and *Prf*^−/−^ mice undergoing transplantation of WT or *PRF1* gene-corrected CD8 T cells all showed only a slight loss of weight before full recovery (Fig 4, *D* and *E*). Kaplan-Meier survival curves show 100% in the B6, 5 of 6 in the WT CD8 T cells, and 7 of 8 in the RV PRF CD8 T cell–treated cohorts (Fig 4, *E*). All surviving mice, including treatment cohorts, were killed at day +30 for further analysis.

Numbers of splenic CD8 T cells and gp33-specific CD8 T cells were increased in LCMV-infected and untreated *Prf*^−/−^ mice but were found at significantly lower levels in B6 and treated cohorts (Fig 4, *F* and *G*). We also investigated the cytopenic phenotype associated with HLH. In *Prf*^−/−^ mice and GFP only–treated *Prf*^−/−^ mice, blood hemoglobin levels were significantly decreased compared with those in healthy control and noninfected mice. In LCMV-infected B6 or *Prf*^−/−^ mice undergoing transplantation with WT CD8 or *PRF1* gene-corrected CD8 T cells, there was no decrease in hemoglobin levels, and levels were significantly greater than that seen in untreated *Prf*^−/−^ mice (Fig 4, *H*). In this experiment no significant difference in platelet levels between untreated LCMV-infected *Prf*^−/−^ mice and healthy control mice were noted, and therefore the effect of gene transfer on platelet levels could not be determined (data not shown). In addition, histology of the spleen and liver showed changes consistent with hyperinflammation in the untreated mice and GFP only–treated *Prf*^−/−^ mice, most notably significantly disrupted splenic architecture and periportal hepatic infiltrates, which were not displayed in other treated cohorts (Fig 4, *I*).

### Lentiviral T-cell perforin gene transfer into human PBMCs restores cytotoxicity *in vitro*

**Fig 5:**
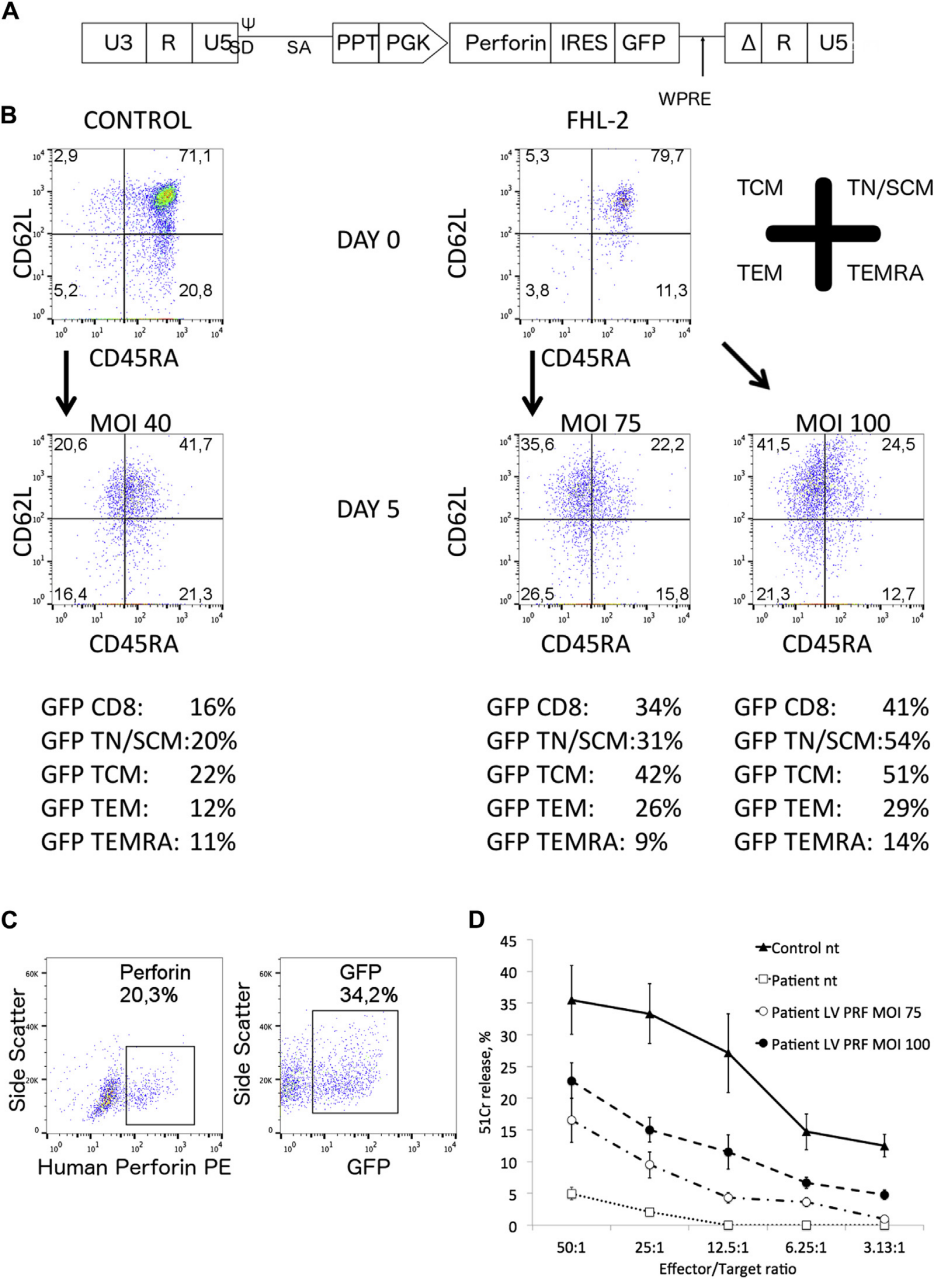
Lentiviral transduction of human T cells. **A,** Schematic representation of the self-inactivating *(SIN)* perforin lentiviral vector. Δ marks SIN deletion with partially deleted U3 of the 3′ long terminal repeat. *ψ*, Packaging signal; *PGK*, phosphoglycerate kinase promoter; *ppt*, central polypurine tract; *SD/SA*, splice donor/splice acceptor; *U3/R/U5*, long terminal repeat elements; *WPRE*, woodchuck hepatitis virus posttranscriptional regulatory element. **B,** Representative CD8 T cell phenotype in a patient with FHL-2 in remission and an age-matched healthy control subject before and 5 days after stimulation (day 2) with different multiplicity of infection *(MOI)*. GFP expression can be verified in all CD8 T cell subsets. Results were gated on CD8 T cells. **C,** Perforin and GFP expression in CD8 T cells in the same patient 72 hours after lentiviral transduction (MOI = 75; for healthy control subjects, see Fig E2, *A*). A transduction efficiency of 34% verified by GFP expression was achieved. Of CD8 T cells, 20.3% express perforin. **D,** Cr^51^ release assay in the same patient showed improved cytotoxicity in the patient transduced with different MOIs. MOI 75 = GFP, 34%; perforin, 20% (Fig 5, *C*); MOI 100 = GFP, 41%; perforin, 25%.

**Fig E2:**
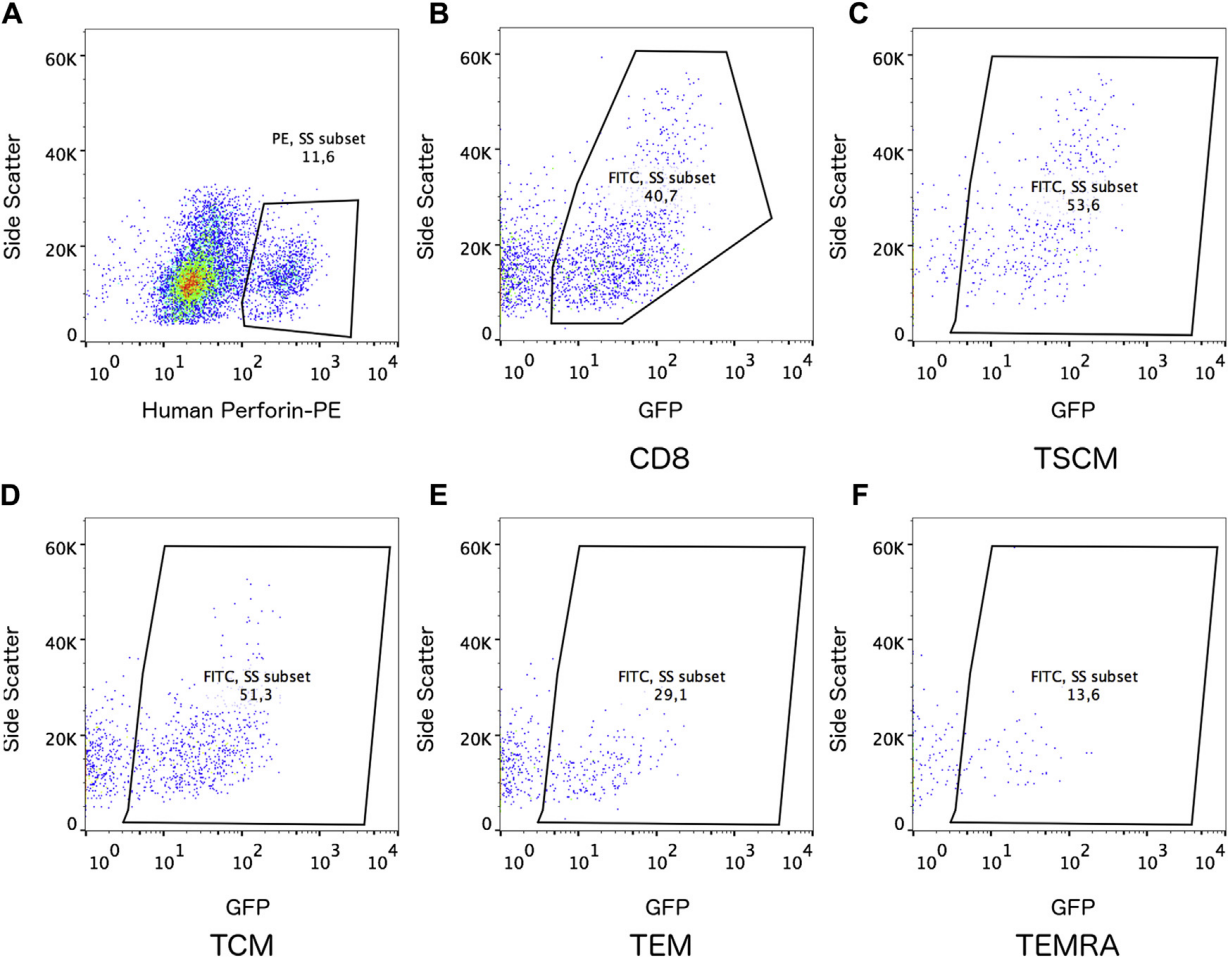
**A,** Representative perforin staining of CD8 T cells in a healthy control subject. **B-F,** GFP expression measured in CD8 T cells and CD8 T-cell memory subsets in the patient with FHL-2 from Fig 5, *B*, at a multiplicity of infection *(MOI)* of 100. (CD8 stem cell memory T *[TSCM]*, TCM, TEM, and effector memory RA T *[TEMRA]* cells).

To assess a possible therapeutic approach, we developed a *PRF1*-expressing lentiviral vector with a phosphoglycerate kinase promoter, as previously described (Fig 5, *A*).^16^ We transduced healthy human PBMCs and were able to transduce all CD8 T cell subsets (CD8 stem cell memory T, TCM, TEM, and effector memory RA T cells) efficiently, as verified based on GFP expression. Similarly, in perforin-deficient patients we were able to transduce CD8 T cells and their subsets (Fig 5, *B*, and see Fig E2, *B-E*, in this article's Online Repository at www.jacionline.org). Interestingly, in both healthy control and perforin-deficient samples, we saw the highest GFP expression in the more naive effector lineages (stem cell memory T and TCM cells). Furthermore, we showed expression of the human perforin protein and GFP using flow cytometry in transduced patient CD8 T cells (Fig 5, *C*). In one patient we were further able to expand cells and assessed functional recovery in a P815 CD3-redirected cytotoxicity on day 10. Interestingly, we found recovery of cytotoxicity, correlating with the multiplicity of infection of lentiviral transduction and transduction efficiency. Untransduced patient cells showed absent cytotoxicity (Fig 5, *D*).

## Discussion

Managing patients with FHL-2 and HLH remains challenging despite novel treatments to suppress the devastating inflammation caused by an environment deficient in cytolytic function. The main pillars of HLH treatment are immune suppression with chemotherapy or serotherapy and subsequent replacement of the hematopoietic compartment. However, not all patients achieve remission, and not all patients have a well-matched donor, leading to a severe increase in morbidity and mortality.^21^ Several novel approaches are being developed, including targeting hypercytokinemia directly. Several studies have shown the pre-emptive or therapeutic efficiency of neutralizing IFN-γ antibodies in the murine model,^14^, ^22^ and phase 2 trials (NI-0501, NCT01818492) are currently ongoing. Furthermore, inhibition of the Janus kinase–signal transducer and activator of transcription pathway and ST2 and IL-33 signaling has been shown to ameliorate the disease in *Prf*^−/−^ mice challenged with LCMV.^23^, ^24^, ^25^

Another approach might be cellular therapy. We targeted CD8 T cells for correction because they are the major effector cell population deficient in perforin-deficient HLH, and effector function failure leads to hyperinflammation and hypercytokinemia. Previous models have shown inability to clear antigen-presenting cells as the main contributing trigger of an increased immune response.^17^ In line with the reported transplantation of WT CD8 T cells into *Prf*^−/−^ mice, we transplanted gene-corrected autologous CD8 T cells to determine functional correction.^15^

In contrast to our progenitor cell approach,^16^ we chose to use a gammaretroviral vector for efficient transduction of murine CD8 T cells given the host restrictions that limit efficient lentiviral transduction of murine T cells.

Although this vector is not in development for clinical use, this was used to show proof of concept that the introduction of *PRF1* into murine CD8 T cells can correct the immune dysregulation. Our reconstitution model proves that corrected autologous CD8 T cells are able to engraft, leading to an equal functional recovery compared with CD8 T cells from mice transplanted with WT CD8 T cells. Use of an LCMV epitope–transfected murine lung carcinoma–based tumor model demonstrates antigen specific *in vivo* functionality. CD8 T cells from P14 mice harboring a defective perforin gene were able to stop tumor formation after transduction of the *PRF1* gene, with similar results *in vitro*. After *in vivo* LCMV infection, which is probably the most testing challenge, the presence of gene-corrected CD8 T cells was able to prevent HLH onset, as demonstrated not only by cytokine and cellular profiles but also more importantly by clinical and survival outcome measures.

Mice were killed before death that occurred after the clinical course of HLH to meet animal license regulations. However, because we have observed the clinical course of numerous other *Prf*^−/−^ mice after LCMV infection and death occurring as an unwanted outcome, we interpret our results as valid survival data. We chose not to cull healthy (ie, WT mice and mice receiving either WT or corrected CD8 T cells) mice at the same time point as mice that had to be killed for clinical reasons. It is possible that the healthy cohort might have been affected at this early time point and that potential anemia and organ destruction could have been restored. However, the clinical appearance of these mice did not suggest HLH at any time point, whereas there were clear abnormalities in the control group.

One surprising observation was that CD8 T cells from *Prf*^−/−^ mice transduced with GFP showed decreased IFN-γ secretion and expansion of tetramer-specific cells. However, the process of transduction confers on these cells a mature (TCM and TEM) phenotype. It is likely that these cells exhibit an anergic phenotype with a decreased capacity for expansion and IFN-γ secretion, and this might confound the IFN-γ results. As a result, there is a significant difference between day 8 IFN-γ levels and CD8 T-and gp33-specific CD8 T cell percentages between unmanipulated *Prf*^−/−^ mice and mice receiving only GFP-transduced CD8 T cells. However, the intervention in the latter group does not confer any clinical protection, as evidenced by increased splenic destruction, poor survival, and cytopenia, which is reflected by similar levels of hemoglobin in both negative control groups. We did not observe any correlation between IFN-γ levels and GFP expression in CD8 T cells in gene-corrected mice (data not shown).

These data suggest that the presence of gene-corrected T cells is able to prevent the onset of HLH after infective triggers, but it still remains unanswered whether these functional T cells are capable of ameliorating the clinical phenotype once dysregulation has already been established in an acute HLH setting. Although anticytokine studies have been performed in mice with an active HLH-like phenotype, WT T-cell transplant studies and subsequent LCMV infection, similar to our experiments, have to date only been performed by using a more preventative approach. Acquiring and transducing T cells in the presence of active LCMV infection might be difficult given the activated state of the T-cell population at this time, and we have not attempted to use this approach in *Prf*^−/−^ mice.

To test our approach in a human setting, we used a previously tested lentiviral vector^16^ containing the human phosphoglycerate kinase promoter and were able to transduce T cell–driven PBMCs, including CD8 T cells. In one patient with perforin deficiency, we were able to transduce cells and, after expansion, were able to further correct cytotoxic T-lymphocyte cytotoxicity.

HLH therapy consists of T cell–suppressing agents; therefore, for a therapeutic approach, the time point of sample collection has to be investigated carefully. There are no studies (as discussed for murine studies) to determine whether T cells in the therapy-naive hypercytokinemic environment are more or less prone to efficient transduction. We were able to transduce patient samples before and during HLH treatment, but as expected, we were not able to find and transduce T cells of severely ill patients who received ATG, for example, as part of the therapeutic regimen (data not shown). On the other hand, etoposide has been shown to have a highly specific effect for activated T cells only while sparing quiescent T cells and innate immune cells in the murine perforin model.^26^ We demonstrate here that we are able to transduce CD8 T cells in 1 patient in remission after receiving etoposide, dexamethasone, and cyclosporine.

The main question remains how to apply corrected T cells in the disease setting. It remains unclear whether we can collect, efficiently transduce, and reinfuse hyperactivated autologous gene-modified T cells to induce HLH remission. We consider that it is more realistic to achieve full or partial remission through conventional means, collect and gene modify T cells, and then reinfuse to correct the defective T-cell compartment. Given the phenotype of the transduced cells with high levels of gene correction in central memory and stem cell memory populations, it is possible that gene-corrected T cells can remain in the circulation for many years. In the context of a clinical trial, we would also be able to see whether these engrafted cells can clear viral challenges without undergoing exhaustion and can maintain a long-term memory profile.

The other question is the level of T-cell engraftment required to protect against HLH. Previous experience in transfer of WT or lentivirus-corrected *Prf*^−/−^ bone marrow in the murine model, as well HSCT results in patients with HLH, suggests that engraftment of 20% donor (or autologous functional) perforin is enough to achieve protection from HLH.^15^, ^16^, ^27^ However, in our studies using gene-corrected CD8 T cells, we saw that 8% to 15% engraftment at the time of LCMV infection was able to protect against LCMV-induced HLH. It is likely that there is a significant expansion of this population at the time of infection, and these data argue that a level of less than 20% might also be applicable in the clinical setting.

The correction of NK cells or other cell types is not addressed by this T-cell approach, and this might have implications for complete disease protection. However, the data in terms of protection from LCMV-induced HLH murine models are very clear that CD8 T cells can protect fully against disease, and we have used this as the basis for this approach. Also, growing experience with the use of gene-modified T cells in cancer immunotherapy makes this approach amenable to rapid clinical translation.

We have now shown in 2 separate studies that gene-modified hematopoietic stem cells or peripheral T cells can restore effector cell cytotoxicity and also protect against LCMV infection in perforin-deficient mice. The safety and efficacy of the use of autologous stem cell and T-cell gene therapy has now been demonstrated in patients with a growing number of monogenic bone marrow diseases^28^, ^29^, ^30^, ^31^, ^32^, ^33^ and acute leukemia.^34^, ^35^ Given the severity of perforin-deficient HLH and the significant morbidities and mortalities associated with current allo-HSCT options, autologous gene therapy with hematopoietic stem or T cells either alone or in combination might be an alternative therapeutic option.

**Key messages**

• Gene-corrected murine *Prf*^−/−^ CD8 T cells engraft efficiently in *Prf*^−/−^ mice and lead to functional *in vitro* recovery.
• CD8 T-cell gene therapy allows *in vivo* protection from tumor challenge and immunopathology in perforin deficiency.

## Methods

### Description of groups

#### Fig 1, *D*: *In vitro* cytotoxicity

• CD8 neg: Splenic CD8 T cells from *Prf*^−/−^ mice, untransduced.
• CD8 RV: Splenic CD8 T cells from *Prf*^−/−^ mice, transduced with gammaretroviral PRF-GFP vector.
Spleens from both groups were pooled from 4 *Prf*^−/−^ mice, and the Cr^51^ assay was done in triplicate.
• CD8 WT: Splenic CD8 T cells from B6 mice, untransduced; spleens were pooled from 2 B6 mice.

#### Fig 2: Reconstitution experiments

• Tx: RV-PRF-CD8 — 17 *Prf*^−/−^ mice received splenic CD8 T cells from *Prf*^−/−^ mice, transduced with gammaretroviral PRF-GFP vector. Fig 2, *A*: Sixteen of 17 mice could be measured. Fig 2, *B*: Ten of 17 mice could be measured. Fig 2, *C*: Eleven of 17 mice could be measured.
• Tx: WT CD8 — 11 *Prf*^−/−^ mice received splenic CD8 T cells from B6 mice; 5 of these 11 mice received splenic CD8 T cells, transduced with gammaretroviral PRF-GFP vector (shown in Fig 2, *A*). Fig 2, *B*: Seven of 11 mice could be measured. Fig 2, *C*: Eight of 11 mice could be measured.
• WT: Four WT mice without any intervention. Cytotoxicity (Fig 2, *B*) and IFN-γ levels (Fig 2, *C*) were measured.
• PRF−/− and Tx: PRF−/− CD8 T cells — 7 *Prf*^−/−^ mice served as a negative control. Four of 7 *Prf*^−/−^ mice did not receive any intervention and 3 of 7 mice received splenic CD8 T cells from *Prf*^−/−^ mice, transduced with gammaretroviral empty GFP vector. Cytotoxicity (Fig 2, *B*) and IFN-γ levels (Fig 2, *C*) were measured.

#### Fig 3: Tumor experiments

Fig 3, *A*: Pooled splenic CD8 T cells from 4 p14 or 4 p14/*Prf*^−/−^ mice. Cytotoxicity was measured in triplicate.

• CD8 P14^+^/PRF−/− RV GFP: Splenic CD8 T cells from p14/*Prf*^−/−^ mice after 3 days of activation and prior transduction with gammaretroviral empty GFP vector.
• CD8 P14^+^/PRF−/− nt: Splenic CD8 T cells from p14/*Prf*^−/−^ mice after 3 days of prior activation but without any transduction
• CD8 P14^+^/PRF−/− RV PRF: Splenic CD8 T cells from p14/*Prf*^−/−^ mice after 3 days of activation and prior transduction with gammaretroviral PRF-GFP vector.
• CD8 P14: Mice received splenic CD8 T cells from P14 mice after 3 days of prior activation.

Fig 3, *C*: All investigated mice were *Prf*^−/−^ mice in which tumor cells were injected.

• PRF−/− (n = 13): Mice did not have any transplantations.
• PRF−/−, CD8 P14 (n = 12): Mice received splenic CD8 T cells from P14 mice after 3 days of prior activation.
• PRF−/−, CD8 P14/PRF−/− RV PRF (n = 9): Mice received splenic CD8 T cells from p14/*Prf*^−/−^ mice after 3 days of activation and prior transduction with gammaretroviral PRF-GFP vector.
• PRF−/−, CD8 P14/PRF−/− nt (n = 5): Mice received splenic CD8 T cells from p14/*Prf*^−/−^ after 3 days of prior activation but without any transduction.

#### Fig 4: LCMV experiments

• PRF−/− (no LCMV): Ten *Prf*^−/−^ mice, not transplanted, not infected, negative control.
• PRF−/−: Six *Prf*^−/−^ mice, not transplanted, infected with LCMV.
• RV GFP CD8 into PRF−/−: Four *Prf*^−/−^ mice, receiving splenic CD8 T cells from *Prf*^−/−^ mice, transduced with gammaretroviral empty GFP vector, infected with LCMV.
• RV PRF CD8 into PRF−/−: Eight *Prf*^−/−^ mice, receiving splenic CD8 T cells from *Prf*^−/−^ mice, transduced with gammaretroviral PRF-GFP vector, infected with LCMV.
• WT CD8 into PRF−/−: Six *Prf*^−/−^ mice, receiving splenic CD8 T cells from B6 mice, infected with LCMV.
• B6: 9 B6 mice, infected with LCMV.
All experiments were done repeatedly, and total numbers shown are the accumulation of all experiments.

#### Clinical information on the patient with FHL-2 in Fig 5

The patient had primary HLH with a central nervous system manifestation at age 1 month. He was treated according to the HLH-2004 protocol. Although receiving treatment with cyclosporine, the boy's renal function was severely compromised but reversible after discontinuation of cyclosporine. The patient received the last etoposide dose 2½ months before blood draw. He had not received steroids for 2 months or cyclosporine for 1 week but had received low-dose hydrocortisone for 2 months and 6-mercaptopurine for 1 month when blood was taken. Four weeks later, he experienced another reactivation, which was again suppressed with etoposide and dexamethasone before undergoing HSCT. The child is now 2½ years old.
